# Mixed-methods investigation into the prevalence, patterns and determinants of prisoner self-harm during the COVID-19 pandemic in England and Wales compared with prepandemic self-harm (COPE)

**DOI:** 10.1136/bmjph-2024-002392

**Published:** 2025-11-21

**Authors:** Kerry Gutridge, Matthias Pierce, Auden Edwardes, Louise Robinson, Emma Plugge, Paula Harriott, Jenny Shaw, Kathryn M Abel

**Affiliations:** 1 Centre for Mental Health and Safety, The University of Manchester Faculty of Biology Medicine and Health, Manchester, UK; 2 Centre for Women’s Mental Health, The University of Manchester Faculty of Biology Medicine and Health, Manchester, UK; 3 Lancashire and South Cumbria NHS Foundation Trust, Preston, UK; 4 Department of Primary Care, Population Sciences and Medical Education, University of Southampton Faculty of Medicine, Southampton, UK; 5 Health and Justice Team, Alcohol, Drugs, Tobacco & Justice Division, Public Health England, London, UK; 6 CEO, UNLOCK National Association of Ex-Offenders Ltd, Maidstone, UK; 7 Greater Manchester Mental Health NHS Foundation Trust, Manchester, UK

**Keywords:** COVID-19, Mental Health, Public Health

## Abstract

**Introduction:**

COPE investigated the prevalence, patterns and determinants of prisoner self-harm during the COVID-19 pandemic, compared with pre-pandemic. The project used the natural experiment of the pandemic to understand effects of prison environments on self-harm.

**Methods:**

COPE was a mixed-methods study involving all prisons within England and Wales. Participants included 80 437 prisoners identified from routine data sources, 71 (ex)prisoners consulted and 89 staff interviewed (average experience: 18.5 years). The main outcome measure was the rate of self-harm incidents reported. Secondary measures included qualitative assessments of prisoner and staff experiences related to self-harm during the pandemic. Qualitative outcomes were derived from thematic grouping of inmate consultation responses and framework analysis of staff responses. The exposure was the pandemic period from March 2020 to December 2021.

**Results:**

There were gender differences in prisoner self-harm rates during the pandemic. In women’s prisons, the pandemic was associated with a 7% overall increase in self-harm (95% CI 2% to 13%) and a 43% increase in self-harm requiring hospitalisation (11% to 83%). Conversely, men’s prisons saw an 8% self-harm decrease (6% to 11%) and a 21% decrease in self-harm hospitalisation. Policy interventions, for example, in-cell telephones and additional phone credit were linked to reduced self-harm in men’s prisons. Introducing video visits was associated with an increase in self-harm in women’s and men’s prisons. Qualitative analysis revealed a complex interplay of factors influencing these outcomes, including tension between the risk of mental health deterioration because of greater isolation and the perceived safety benefits of a restricted regime.

**Conclusions:**

The pandemic, and policy changes, were associated with changes in prisoner self-harm. Distinct patterns emerged between genders underscoring the need for gender-specific prison policies, especially during crisis management. Furthermore, the complex relationship between policy implementation and self-harm highlighted the importance of tailoring prison environments to effectively address the population’s diverse needs.

**WHAT IS ALREADY KNOWN ON THIS TOPIC**

- Prisoners have very high levels of self-harm compared with the general population.
- Currently, relatively little is known about how changes to the prison environment influence self-harm.

**WHAT THIS STUDY ADDS**

- Using the natural experiment of the pandemic, the COVID-19 measures and prisoner self-harm (COPE) study identified modifiable environmental factors which influence self-harm. In cell phones, extra phone credit and changes to the incentive scheme were associated with a reduction in self-harm (for one or both sexes); however, video visits were associated with an increase in self-harm.

**HOW THIS STUDY MIGHT AFFECT RESEARCH, PRACTICE OR POLICY**

- Our findings suggest that prisoner self-harm rates can be influenced (positively and negatively) by the prison environment, suggesting potential targets for intervention and policies which merit further consideration and adaptation.

## Introduction

 In March 2020, at the start of the COVID-19 pandemic, Public Health England modelled the impact on prisons, predicting there could be 2700 COVID-19-related deaths if actions were not taken to minimise transmission.^1^ Prisons are high-risk environments for COVID-19 infection because of overcrowding, poor sanitation, inadequate ventilation and prison-to-prison/community-to-prison movement of staff and prisoners.^2 3^ Moreover, prisoners themselves are at a particular risk of severe morbidity and death from COVID-19 because of pre-existing health vulnerabilities.^4^ To minimise transmission and prepare for staff shortages, prisons introduced an Exceptional Delivery Model whereby social visits ceased, along with education and training, non-essential employment, gym access, and religious and general association.^1^ Prisons also limited the number of people out of their cells at any one time to take part in activities such as exercise, showering and telephone calls.

Early on, there were concerns that such isolation might worsen prisoner mental health and self-harm rates,^5^ which were already considerably worse than in the general population.^6^ In 2019, the number of recorded self-harm incidents in prisons was at a record high of 63 328, up 12% from the previous 12 months.^7^ Currently, relatively little is known about how changes to the prison environment influence self-harm.^8^ However, solitary confinement, for discipline or personal protection, is a risk factor.^9^ During the pandemic, prisoners self-reported that isolation increased their thoughts of self-harm and suicidal ideation^10^ and that self-harm was being used to cope with the effects of confinement.^11^ Ministry of Justice (MOJ) data suggested, overall, self-harm reduced during the pandemic.^12^ This MOJ dataset does not include a prepandemic comparison that accounts for pre-existing trends; neither does it examine variation between prisons, by gender, or at different time periods during the pandemic; nor the impact of any mitigation policies designed to minimise the effects of social isolation, for example, video calls for prisoners.

We conducted a mixed-methods study to ascertain individual, environmental and policy factors associated with self-harm changes in prison during the pandemic. The research was designed to provide evidence on self-harm to guide self-harm policy. Our primary aim was to determine whether the rate of self-harm in prison changed during the pandemic, taking existing trends into account. We also aimed to determine whether COVID-19 mitigation or well-being policies, instigated in prisons during the pandemic, had a positive or negative effect on prisoner self-harm. We triangulated quantitative analyses with qualitative consultation responses from prisoners and interviews with staff and used this to understand the mechanisms behind any change.

## Methods

### Study design and setting

This study used a convergent mixed-method design, whereby quantitative data and qualitative data were analysed separately and then results were integrated to form robust conclusions. There were three sources of data: (1) longitudinal quantitative data on self-harm or prison characteristics from routinely collected sources; (2) quantitative data on COVID-19 related mitigation policies from a survey, administered to prison staff and (3) qualitative data from written and telephone consultation with prisoners and ex-prisoners and interviews with staff.

### Data sources and data collection

#### Routine data on self-harm

In England and Wales, data are routinely collected by the MOJ and His Majesty’s Prison and Probation Service (HMPPS) on safety information, health outcomes, prisons characteristics and prisoner demographics. During the pandemic, additional data were collected by the MOJ and HMPPS on COVID-19 factors including the level which a prison was in the national COVID-19 preparedness framework, whether a prison recorded an outbreak and the number of positive tests. Relevant data were extracted from these sources by the MOJ/HMPPS and provided to the research team for March 2018 to December 2021. The routine data on self-harm events relied on staff report, and therefore missing cases, or instances where the staff member did not input it to the system, are not captured. Characteristics of prisons and prisoners are routinely collected and assumed to be complete.

Additionally, every prison in England and Wales was approached to participate in a telephone survey between May 2022 and July 2022. With consent, the telephone call was audio-recorded using an encrypted external recording device. Prison staff provided data on prison-level mitigations or policies that might have influenced self-harm during the pandemic. These were retrospectively recalled by staff, who indicated whether a policy had been implemented and, if so, when. An interview was also conducted with staff to collect qualitative data on their perceptions of how these measures affected their work, the functioning of the prisons and the impact they might have had on prisoner well-being and self-harm. Qualitative data were also collected from a written and telephone consultation with prisoners and ex-prisoners from the Prison Policy Network by the Prison Reform Trust, to ascertain the extent to which the pandemic affected prison life and prisoner well-being. There was some missing or incomplete information on pandemic-related policies from 24 prisons (21%), of which three were female (25%) and 21 were male (21%).

#### Prison staff survey

##### Sample

For phase 2 of the research, the staff survey, we aimed to interview one member of staff, with the requisite knowledge of policy changes during the pandemic, from each of the 112 prisons in England and Wales. We determined which members of staff had the required knowledge of prison safety policy in conversation with the prison governor. The staff member did not need to have a pre-specified role in the prison and the governor was eligible to participate.

###### Inclusion criteria

- Prison staff who are familiar with the individual prison’s response to the pandemic, in terms of safety.
- Prison staff who have worked during the pandemic.

###### Exclusion criteria

- Staff who are not familiar with the prison’s safety response.
- Prison staff who have not worked during the pandemic.

### Consultation

For the consultation, we invited all members of the Prison Policy Network, a national network of over 1000 prisoners and ex-prisoners, to provide a response. The following criteria were used:

#### Inclusion criteria

- Current prisoners or ex-prisoners who were in prison during the pandemic in England/Wales.

#### Exclusion criteria

- People who have not experienced living in prison during the pandemic.

### Outcome

The primary outcome was rate of self-harm and the secondary outcome was self-harm requiring hospitalisation. Self-harm events were defined as incidents including, but not limited to, cutting, overdose or hanging, as per the criteria used in the safety in custody statistics reported by English and Welsh prisons.^13^ Self-harm reporting in prisons involves a standard protocol, where incidents are logged onto IT systems by a staff member.

### Exposures

Exposure to the COVID-19 pandemic was defined as the period from March 2020 to December 2021, the end of data collection for the study. Additional exposures, collected from routine data, were the level the prison was in the National Framework (which reflects the severity of the restrictions imposed in individual prisons), and whether there was a COVID-19 infection outbreak in a prison during that month. COVID-19 policy variables, collected from the staff survey, were checked for completeness and whether the timing of the policy could be reliably recalled. Policies of sufficient quality, assessed based on reliable recall of the timing, included in the analysis were:

- In-cell telephony, where a phone was installed in prison cells.
- Suspending in-person visits, where all visits from family or friends outside the prison were suspended to stop the virus from spreading.
- Additional phone credit, where prisoners in the men’s estate were given £5 a week extra phone credit. Those in the women’s estate were given £10 a week, in recognition that they were being disproportionately affected by restrictions on contact with families.
- Starting video visits where prisoners could talk to family and friends via video-link. These are called purple visits in the prison estate as that is the name of the company providing the visitation platform.
- Suspending ‘basic regime’ incentive level; meaning suspension of the most restrictive level of the Incentives Earned Privileges process, which is designed to incentivise good behaviour, in which privileges (eg, in-cell television (TV)) are stopped.
- Administering a well-being fund, where £10 per prisoner was paid to prisons to spend on prisoner well-being.

### Quantitative data analyses

The self-harm rate was estimated as the number of self-harm events, in a month, per 100 prisoners. The effect of the pandemic on self-harm and self-harm requiring hospitalisation was estimated using Poisson regression models, fitted separately to data from women and men’s prisons, with a random effect for each prison to account for prison-level clustering, and an offset term for the number of prisoners in each month. Models included a categorical variable indicating if the month was during the pandemic. In addition, they included variables for calendar month and calendar month squared to account for secular trends in self-harm. These models estimated risk ratios (RRs), estimating relative changes in self-harm associated with each exposure, accounting for underlying trends and seasonality.

Data from the staff survey were linked to each prison to determine the timing of individual policy changes. A binary variable was created for each, equal to one if the policy was in place for at least half the month and zero otherwise. This was included in separate regression models, using the same specification as above, but with the additional variable to indicate ‘phase of the pandemic’. Those prisons (n=24) with missing or incomplete information from this survey were excluded from this analysis.

### Qualitative data analysis

The Prison Policy Network consultation responses were grouped thematically, according to the research questions, by the Prison Reform Trust, and a summary report provided to the research team (available on request). Free text data from the prison survey and staff interviews were transcribed and analysed using Framework Analysis.^14^ Transcripts were shared between four researchers who familiarised themselves with the data and completed preliminary coding. This coding was then reviewed by the qualitative lead (KG) who constructed an initial thematic framework in discussion with the researchers who completed the survey and interviews. Once the framework had been created, the remaining transcripts were allocated to two researchers who completed line by line indexing of the data within the framework. If new themes emerged, these were incorporated into the framework. The qualitative lead (KG) then engaged in data interpretation, together with experts-by-experience, identifying linkages between the different aspects of the data and explanations for patterns within the framework. Triangulation and interrogation of the data with a panel of key stakeholders were used to establish trustworthiness.

Once the qualitative and quantitative data were analysed, the findings were compared, examining areas where results converge, diverge or complement each other.

### Patient and public involvement

Consultations were carried out with prisoners and ex-prisoners throughout to ensure interpretation of our findings incorporated prisoner views. Additionally, the research team included the head of engagement from the Prison Reform Trust and two further experts-by-experience who contributed to the research design and interpretation of the analysis.

## Results

All prisons in England and Wales were included in the quantitative analysis, 11 from the women’s estate, 100 from the men’s estate and 1 that included both women and men (housed in separate wings; table 1). Men’s prisons tended to be larger (median of 691 prisoners vs 307 in women’s) and more likely to have a higher proportion of prisoners in crowded cells (10% vs 1%).

**Table 1:** Description of prisons in the sample

Statistics*	Women’s estate	Men’s estate
Number of prisons†	12	101
Number of prisoners	3627	76 810
Median size of prison (IQR)	307 (282, 369)	691 (514, 1012)
% of prisoners in each prison, median (IQR):		
In crowded cells	1.0 (0.0, 8.0)	10 (0, 33.7)
Remand prisoners	17.6 (13.5, 25.0)	0.6 (0.2, 31.2)
Ethnic minority prisoners (excluding white minorities)	18.6 (9.1, 21.3)	22.6 (16.3, 36.9)
Prisoners <25 years	7.9 (6.3, 12.2)	13.1 (7.1, 17.9)
Prisoners >50 years	3.1 (1.0, 4.3)	3.8 (1.9, 7.0)

* Statistics were measured using March 2020 data.

† The total number of prisons is 113 because one prison (HMP Peterborough) holds both men and women.

The survey and interview were completed with a nominated staff member from 89 prisons (79% of the prisons). The characteristics of the staff that were interviewed are shown in online supplemental table S1. In total, we received 89 responses to the Prison Policy Network consultation from 71 individuals, representing 32 different prisons. Of those 89 responses, 38 were via ‘email a prisoner’, 43 via postal letters and 8 were by telephone contributions. Demographic information on PPN members is not routinely collected by the Prison Reform Trust to ensure anonymity.

### Association between the pandemic and self-harm

#### Findings from the quantitative analysis

**Figure 1:**
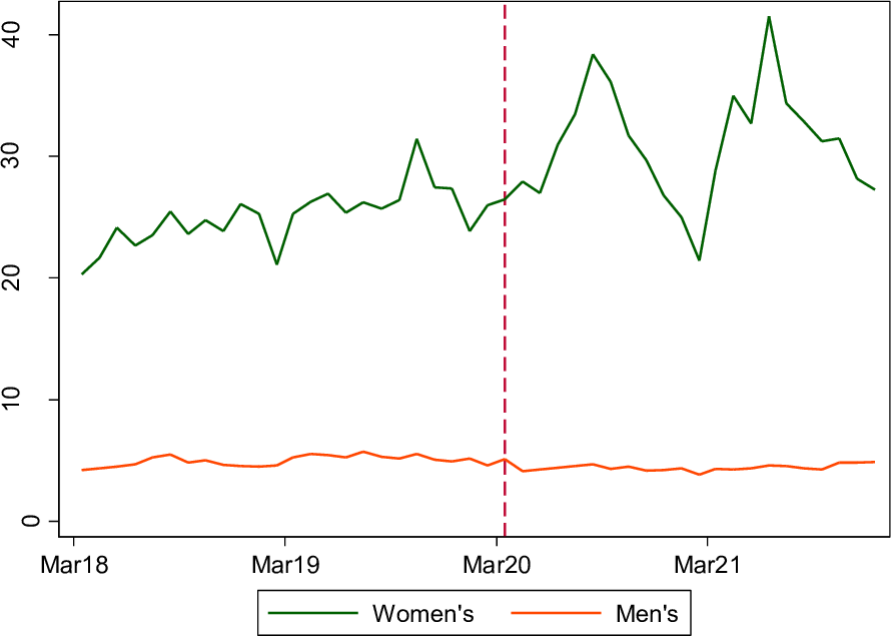
Monthly rate of self-harm, per 100 prisoners, men’s and women’s estate.

Accounting for prepandemic trends, in the women’s estate the pandemic was associated with a small 7% increase in self-harm (95% CI 2% to 13%; figure 1, table 2); and with a small (8%) decrease (95% CI 6% to 11%) in the men’s estate. Self-harm requiring hospitalisation showed the same direction of effects, but more pronounced (online supplemental figure S1): in the women’s estate, the pandemic was associated with a 43% increase (95% CI 11% to 83%), and in the men’s estate, a 21% decrease (95% CI 12% to 28%), compared with the strictest applied stage of lockdown in the national COVID-19 response framework (level 4), less-strict lockdowns in the women’s estate were associated with reduced rates of self-harm, but increased rates of self-harm requiring hospitalisation (table 2). COVID-19 outbreaks were also associated with a lower rate of self-harm, but more self-harm requiring hospitalisation. In women’s prisons, having a higher proportion of shielding prisoners was associated with a 20% increase in self-harm. In men’s prisons, there was no clear evidence of change in self-harm during COVID-19 outbreaks or different stages of the COVID-19 National Framework.

**Table 2:** Association between pandemic exposures and self-harm, in men’s and women’s estates

Outcome	Exposure	Women’s estate			Men’s estate		
		Rate*	Rate ratio (95% CI)	P value	Rate*	Rate ratio (95% CI)	P value
Self-harm	Before pandemic	265.2	Ref	<0.001	52.5	Ref	<0.001
	During pandemic	307.9	1.07 (1.02 to 1.13)		44.5	0.92 (0.89 to 0.94)	
	Level in the national framework						
	Stage 4	297.3	Ref	<0.001	44.8	Ref	<0.001
	Stage 3	354.5	1.40 (1.33 to 1.48)		46.0	1.04 (1.01 to 1.07)	
	Stage 2	292.2	1.39 (1.28 to 1.50)		49.2	1.09 (1.05 to 1.13)	
	Stage 1	218.4	1.25 (1.11 to 1.41)		30.2	0.95 (0.89 to 1.01)	
	Outbreak of COVID-19						
	No	310.2	Ref	<0.001	44.3	Ref	0.209
	Yes	302.0	0.91 (0.88 to 0.94)		46.6	0.99 (0.97 to 1.01)	
	Shielding prisoners*	–	1.20 (1.01 to 1.43)	0.038	–	0.99 (0.97 to 1.01)	0.390
Self-harm requiring hospitalisation	Before pandemic	6.4	Ref	0.005	3.3	Ref	<0.001
	During pandemic	7.6	1.43 (1.11 to 1.83)		2.5	0.79 (0.72 to 0.88)	
	Level in the national framework						
	Stage 4	8.5	Ref	0.010	2.5	Ref	0.816
	Stage 3	6.8	0.68 (0.51 to 0.90)		2.7	0.96 (0.85 to 1.10)	
	Stage 2	6.0	0.55 (0.38 to 0.80)		2.6	0.93 (0.79 to 1.09)	
	Stage 1	3.0	0.53 (0.25 to 1.12)		2.3	0.92 (0.72 to 1.19)	
	Outbreak of COVID-19						
	No	6.6	Ref	<0.001	2.5	Ref	0.586
	Yes	10.2	1.49 (1.24 to 1.81)		2.6	0.98 (0.92 to 1.05)	
	Shielding prisoners*	–	1.48 (0.45 to 4.89)	0.518	–	0.92 (0.81 to 1.05)	0.205

* Shielding described the separation of clinically vulnerable prisoners from the general population. Analysis was restricted to pandemic months only.

In the women’s estate, prisons with a higher proportion of ethnic minority (excluding white minority) prisoners saw a greater increase in self-harm associated with the pandemic than those with a lower proportion (table 3). Also, in women’s prisons, those with more prisoners in crowded cells responded more poorly to the pandemic, and in men’s and women’s prisons, having a larger prison predicted a worse response to the pandemic. In men’s prisons, there was a clearer improvement associated with the pandemic in prisons with the lowest HMPPS performance rating^15^ (RR 0.81, 95% CI 0.77 to 0.84) compared with prisons with the highest rating.

**Table 3:** Association between the pandemic and self-harm within particular subgroups, defined by prison characteristic

Prison characteristic	The association between the pandemic and self-harm			
	Women’s estate		Men’s estate	
	RR (95% CI)	P value*	RR (95% CI)	P value*
Proportion of ethnic minority (excluding white minority) prisoners		<0.001		<0.001
≥25%	1.52 (1.41 to 1.63)		0.93 (0.90 to 0.95)	
<25%	1.00 (0.96 to 1.05)		0.90 (0.88 to 0.92)	
Proportion of prisoners between ages of 18 and 24		0.738		0.227
≥10%	1.02 (0.95 to 1.11)		0.92 (0.90 to 0.95)	
<10%	1.04 (1.00 to 1.08)		0.91 (0.89 to 0.94)	
Open prisons/Cat D		0.894		0.164
Yes	1.04 (0.62 to 1.74)		0.91 (0.89 to 0.94)	
No	1.07 (1.02 to 1.13)		0.98 (0.89 to 1.08)	
Proportion of prisoners in crowded cells		<0.001		0.542
<10%	1.06 (1.01 to 1.12)		0.91 (0.89 to 0.94)	
≥10%	1.12 (1.05 to 1.19)		0.92 (0.89 to 0.94)	
Size of prison		<0.001		0.006
<500 prisoners	0.98 (0.93 to 1.03)		0.86 (0.82 to 0.89)	
≥500 prisoners	1.80 (1.66 to 1.96)		0.93 (0.90 to 0.95)	
Proportion of prisoners on remand		<0.001		0.099
<5%	1.10 (1.04 to 1.15)		0.93 (0.91 to 0.96)	
≥5%	0.85 (0.78 to 0.93)		0.84 (0.81 to 0.87)	
Prison rating		0.1826		<0.001
1 (lowest)	–		0.81 (0.77 to 0.84)	
2	–		0.96 (0.93 to 0.99)	
3	1.08 (1.03 to 1.13)		0.90 (0.88 to 0.93)	
4 (highest)	0.98 (0.84 to 1.13)		0.99 (0.92 to 1.05)	

* P value testing for effect modification; that is, the null hypothesis is that there is no difference in RR within levels of the variable. Note that, when testing for effect modification, continuous variables were kept as continuous; however, for presentation purposes, they are included as binary variables, split at (or near) the median level.

RR, risk ratio.

#### Findings from the qualitative analysis

Prisoner consultation found prisoners reported deteriorating mental health with experiences of low mood and anxiety about COVID-19, both for themselves and their families; as well as feelings of hopelessness, frustration, social isolation and a lack of communication. For example:

I was worried about myself and Covid and I was worried about my family and Covid and there was no one to talk to; prison life just stopped as if we were being mothballed or put in a coma with no end date.

However, a frequent theme in the staff responses was that some people in prison felt better during COVID-19 restrictions because they felt safer.

And I think when we surveyed the prisoners during COVID they actually said they felt safer in smaller groups. And obviously if you feel under threat, that’s also a trigger for self-harm. So, if less people were actually feeling under threat, then I think that’s the explanation for why self-harm in this prison [went down]

According to staff, feelings of increased safety were attributed to prisoners socialising in smaller groups when the regime allowed it, leading to more social cohesion, less access to drugs, less bullying, less violence and, as a result, less self-harm. This was corroborated by some people in prison, who felt the more restricted regime was safer. However, these data also highlighted a tension between less exposure to social stress and the need for socialisation, especially if smaller groups meant more time in cell. Staff did not emphasise the effects of more time in cell on mental health as much as prisoners did. In the prisoner consultation, participants reported that the lack of social interaction was detrimental to their mental health and hindered their personal development. They also reported that new prisoners were a group that initially felt safer in the more restricted regime, but this was not beneficial in the longer term:

In general, those that are new to the prison system, or are scared of other prisoners, or are generally nervous are pleased with the routine of bang up as they feel it keeps them safe. But it isn’t good for them in the long-term including upon release; isolation and poor social skills are often factors in offending.

Prisoners also expressed concern about staff wanting to maintain the restricted regime, indicating a breakdown of trust between prisoners and staff:

Now there is a widespread belief on the part of prisoners that staff, on the whole, are very keen to maintain a ‘bare bones’ regime and want to make many features of lockdown permanent.

### Association between pandemic-related policies on self-harm

#### Findings from the quantitative analysis

In-cell telephones were associated with a reduction in the rate of self-harm: by 17% in women’s prisons (95% CI 14% to 20%); and by 2% in men’s prisons (95% CI 1% to 4%; table 4). Having additional phone credit was associated with a 17% reduction in men’s prisons, but with no change in women’s. Suspending in-person visits was associated with a small 6% reduction in men’s (95% CI 4% to 8%); but not women’s prisons. Suspending basic level of the Incentives and Earned Privileges scheme was associated with a 22% decline in self-harm in men’s prisons (95% CI 18% to 26%). Counter to expectation, having Purple Visits was associated with an increase in self-harm in both women and men’s prisons. Also, counter to expectation, while the well-being Fund was associated with a 12% reduction in self-harm in women’s prisons (95% CI 6% to 18%), it was associated with a 31% increase in men’s prisons (95% CI 27% to 36%).

**Table 4:** Association between prison-specific policies and self-harm

Policy	Women’s prisons		Men’s prisons	
	RR (95% CI)*	P value	RR*	P value
In-cell telephones	0.83 (0.80 to 0.86)	<0.001	0.98 (0.96 to 0.99)	0.003
Additional phone credit	1.00 (0.91 to 1.10)	0.936	0.83 (0.80 to 0.86)	<0.001
Suspended in-person visits	1.04 (0.98 to 1.10)	0.203	0.94 (0.92 to 0.96)	<0.001
Purple visits	1.14 (1.05 to 1.24)	0.002	1.07 (1.04 to 1.11)	<0.001
Suspending basic regime	1.00 (0.91 to 1.10)	0.957	0.78 (0.74 to 0.82)	<0.001
Well-being fund	0.88 (0.82 to 0.94)	<0.001	1.31 (1.27 to 1.36)	<0.001

* RR’s from fixed effects models, controlling for month (linear and squared terms) and the phase of the pandemic (indicator variable).

RR, risk ratio.

#### Findings from the qualitative analysis

Both staff and prisoners were positive about in-cell telephones, suggesting they allowed more family contact and continued health checks.

We had phones in the cell in the prison I was in at the time, and that was an absolute godsend, but here (category C prison), we have some wings with communal phones on the landings, no way to call for more than 15 minutes at a time. I don’t know how I would have coped without the in-cell phone at the time.

Staff suggested extra phone credit helped reduce self-harm in the men’s estate by reducing debt, which is thematically linked to bullying and self-harm.

But by having that extra phone credit and that extra money, it stopped them thinking about, well I need to borrow, or I need to lend vapes or I need to do this, because I’ve got the money there. So those prisoners that normally wouldn’t have any money and wouldn’t go to work because they were under threat of being bullied or getting themselves into debt, we gave them the money to buy what they needed. They never had to borrow or get into debt. And so, in turn, by not getting into debt, not being bullied, self-harm was down.

Qualitative findings did not explain why extra phone credit was not associated with reduced self-harm in the women’s estate. Interview data indicated the suspension of basic level of the Incentives and Earned Privileges process (the most restrictive) was associated with reduced self-harm in men’s prisons. Staff and participants suggested not removing TVs helped reduce self-harm because TV acted as a distraction, reduced anger about TV removal and allowed communication through WayOut TV about COVID-19 measures. Some staff wanted to reintroduce the basic level to enforce boundaries again but mentioned a shift towards more reward-focused rather than punitive measures.

We’ve learnt through Covid that actually taking a telly as a punishment is not helpful to somebody’s mental health, whether they self-harm, whether they take drugs, it’s probably going to make them take drugs more because they’re bored. So, we moved away from that and we’re looking more at a proper incentivised IEP scheme, rather than it being all sanction led.

The qualitative data showed a complex relationship between self-harm and loss of in-person visits: prisoners found not having visits distressing and said it was a reason they or others had self-harmed.

Isolation from family and others on the wing has impacted many in terms of their ability to socialise, concentrate, remain positive.

I self-harmed when I couldn’t get hold of my family when my sister died.

Overall, a mixed picture emerged about in-person and Purple Visits. Staff reflected that face-to-face visits can be distressing, and their removal may have had different effects. In contrast to the quantitative data, staff felt Purple Visits had positive effects:

And that’s when we bought in Purple Visits whereby, we would do video visits on an iPad, which they were very successful. A lot of prisoners were very grateful for them, because, obviously, there was no face-to-face visits going on. So, that was probably a real game changer, because that was the first time then, apart from phone calls, that people were actually seeing their family.

Both prisoners and staff described problems with Purple Visits’ technology which was distressing, such as poor connectivity, video visits cutting out, families struggling with technology and a limited number of devices or staff to facilitate their use. While some liked seeing their home in the background, others found it upsetting.

Some people find it hard, though, because they prefer to be on the phone because they could talk to their family but if they could see their family in the house that made them more homesick.

There were minimal qualitative data about the Wellbeing Fund, with many staff struggling to recall what it was or what it was spent on. Further detail on the qualitative analysis can be found in online supplemental table S2.

## Discussion

### Principal findings

We examined how environmental and policy changes in English and Welsh prisons during the pandemic could be used to identify the determinants of prisoner self-harm. The pandemic had notable gendered differences: self-harm rates increased in women’s prisons but decreased in men’s prisons, with similar patterns for self-harm incidents and hospitalisations. The increase in women’s prisons was particularly marked in crowded and large prisons. Some pandemic-related policies affected self-harm rates: in-cell phones, extra phone credit and suspension of the basic level of the Incentives and Earned Privileges scheme reduced self-harm in men’s prisons, while in-cell phones also reduced self-harm in women’s prisons. Unexpectedly, suspending in-person visits slightly reduced self-harm in men’s prisons, whereas ‘Purple’ (video) visits increased self-harm in women’s and men’s prisons.

The qualitative data supported these findings, providing additional context. Staff suggested that restricted socialising in men’s prisons reduced exposure to self-harm triggers like access to drugs, bullying and violence. However, prisoners reported that social isolation led to low mood, anxiety, hopelessness and frustration, and they worried about its long-term impact on personal development. Both prisoners and staff highlighted the importance of in-cell phones and extra phone credit for maintaining family relationships and reducing isolation. In-cell phones also facilitated continued mental health services and extra phone credit reduced tension and victimisation. The suspension of the basic regime and retaining TV access were seen as calming and distracting. However, video visits could be detrimental, potentially increasing homesickness or causing frustration or disappointment due to technical difficulties.

### Comparison with other studies

Gender differences in prisoner self-harm are well-recognised, with sex differences in the incidence and degree of injury caused.^16^ Our study found that female and male prisoners responded differently to environmental changes during the pandemic. Women in the community were also more susceptible to self-harm during the pandemic than men.^17^ Our findings align with other studies showing that in-cell phones and additional phone credit reduce tension between prisoners by removing competition for limited phones and reducing phone credit debt and victimisation.^18^ Prisoners reported these measures as beneficial during the pandemic for maintaining family contact.^19^

Consistent with other qualitative research,^19^,^21^ we reported that isolation from peers negatively impacted prisoners’ mental health and self-harm. However, for some prisoners, particularly men, reduced social interaction lowered the risk of victimisation, which prior research has highlighted as a strong risk factor for self-harm.^9^ The suspension of in-person visits revealed a complex picture: while it was associated with reduced self-harm in men’s prisons, other studies reported significant distress due to the loss of in-person visits during the pandemic,^21^ and one study suggested that an absence of visits predicted self-harm ideation for women.^22^ Finally, the association between the suspension of the basic regime and reduced self-harm in men’s prisons is revealing. Our qualitative research suggested that retaining access to TV while in cell, typically removed under the ‘basic’ regime, provided a valuable distraction. Future policies might consider a more rewards-based approach in the Incentives and Earned Privileges process.

### Study limitations

We present a unique mixed-method study examining how the pandemic affected prisoner self-harm. There are several strengths; for example, we used data from all prisons, meaning the study has excellent generalisability. However, limitations to our findings should be considered. First, inevitably, self-harm is underreported. This will be a source of bias if the proportion of self-harm that remains unreported is related to the exposure under consideration: in this case, the pandemic. Our consultations alerted us to the fact that prepandemic, self-harm was also reported by external organisations (eg, charities) unable to operate during the pandemic, raising the possibility of under-reporting. However, the ratio of serious to non-serious self-harm events, where the former are less likely to be subject to underreporting,^13^ only changed slightly during the pandemic (in the women’s estate it was 49.9 before the pandemic, and 48.9 afterwards; in the men’s estate it was 19.9 before the pandemic and 19.7 afterwards). Second, despite the longitudinal design, we cannot rule out bias from unmeasured time-varying confounding, for example if the prisoner population included fewer people with substance misuse problems (a self-harm risk factor^23^) during the pandemic. Third, the staff survey was conducted 2 years after the start of the pandemic, by 79% of the prisons, and may have been subject to recall bias. We mitigated this through: prepopulating the survey fixed dates related to the policy; sending the survey in advance so staff could consult their notes and colleagues; and asking staff to email responses to the survey after the phone discussion, once they had been able to gather more information. A fourth limitation was that, under normal circumstances, we would have conducted face-to-face interviews or focus groups with prisoners; however, this was not possible because of COVID-specific research restrictions. Finally, as we did not have individual-level data, we could not account for underlying changes in the prisoner population, nor determine whether observed changes in self-harm reflected shifts in its prevalence across the population or continued high-frequency self-harm among a small number of individuals.

### Conclusions

Prisoner self-harm has been conceived as broadly deriving from factors within the individual^9^ which create substantial challenges for effective intervention. Our findings suggest prisoner self-harm is significantly influenced by modifiable factors within the prison environment and the prison regime; and these factors influence self-harm in some prisons/prisoners more than in others. This not only helps inform thinking about self-harm, but also about how prison environments (eg, socialising regimes) can be part of the solution. In addition, women prisoners continued to be more likely to self-harm during the restricted COVID-19 regime than men. Calls for gender-specific prison policies are not new^24^ but our findings highlight gender-specific areas to focus action: in men, social mixing may be a risk factor, and ensuring the safety of individuals and minimising bullying and victimisation will be important; for women, socialisation is important, as is access to in-cell telephones and this should be facilitated by the prison regime. This provides valuable evidence, not just on planning for future emergencies, but is of relevance to HMPPS Digital, Data and Technology Strategy, the Incentives and Earned Privileges Process, and the Video Call Framework and their potential effects on prisoner self-harm in women and men.

## Supplementary material

10.1136/bmjph-2024-002392 online supplemental file 1

## Data Availability

Data are available on reasonable request. Data may be obtained from a third party and are not publicly available.

## References

[1] Ministry of Justice, PHE, HMPPS (2020). Briefing paper: interim assessment of impact of various population management strategies in prisons in response to COVID-19 pandemic in England. https://www.gov.uk/government/publications/covid-19-population-management-strategy-for-prisons.

[2] Kinner SA, Young JT, Snow K (2020). Prisons and custodial settings are part of a comprehensive response to COVID-19. Lancet Public Health.

[3] SAGE EMG Transmission Group (2021). COVID-19 transmission in prison settings: March 2021. https://www.gov.uk/government/publications/emg-transmission-group-covid-19-transmission-in-prison-settings-25-march-2021.

[4] Beaudry G, Zhong S, Whiting D (2020). Managing outbreaks of highly contagious diseases in prisons: a systematic review. BMJ Glob Health.

[5] Johnson L, Gutridge K, Parkes J (2021). Scoping review of mental health in prisons through the COVID-19 pandemic. BMJ Open.

[6] Fazel S, Baillargeon J (2011). The health of prisoners. The Lancet.

[7] Ministry of Justice (2020). Safety in custody quarterly bulletin: December 2019. https://www.gov.uk/government/statistics/safety-in-custody-quarterly-update-to-december-2019.

[8] Stephenson T, Leaman J, O’Moore É (2021). Time out of cell and time in purposeful activity and adverse mental health outcomes amongst people in prison: a literature review. Int J Prison Health.

[9] Favril L, Yu R, Hawton K (2020). Risk factors for self-harm in prison: a systematic review and meta-analysis. Lancet Psychiatry.

[10] Kim H, Hughes E, Cavanagh A (2022). The health impacts of the COVID-19 pandemic on adults who experience imprisonment globally: A mixed methods systematic review. PLoS ONE.

[11] HMI Prisons (2022). HM chief inspector of prisons for England and Wales annual report 2021-22 HC 411. https://www.justiceinspectorates.gov.uk/hmiprisons/inspections/annual-report-2021-22/.

[12] MOJ (2024). Justice in numbers: public protection. https://data.justice.gov.uk/justice-in-numbers/jin-public-protection#self-harm-rate.

[13] Ministry of Justice (2021). Guide to safety in custody statistics ministry of justice statistics bulletin.

[14] Spencer L, Ritchie J, O’Connor W, Ritchie J, Lewis J, Nicholls CM (2014). Qualitative research practice: a guide of social science students and researchers.

[15] MOJ (2020). Annual prison performance ratings 2019/20. https://www.gov.uk/government/statistics/prison-performance-ratings-2019-to-2020.

[16] Hawton K, Linsell L, Adeniji T (2014). Self-harm in prisons in England and Wales: an epidemiological study of prevalence, risk factors, clustering, and subsequent suicide. The Lancet.

[17] Dubé JP, Smith MM, Sherry SB (2021). Suicide behaviors during the COVID-19 pandemic: A meta-analysis of 54 studies. Psychiatry Res.

[18] McKay C (2022). The Carceral Automaton: Digital Prisons and Technologies of Detention. Int J Crime Justice Soc Democr.

[19] (2020). Independent advisory panel of deaths in custody. “keep talking, stay safe” a rapid review of prisoners’ experience under COVID-19. https://www.iapondeathsincustody.org/news/keep-talking-stay-safe.

[20] User Voice & Queen’s University Belfast (2022). Coping with covid in prison: the impact of the prisoner lockdown. https://www.uservoice.org/consultations/coping-with-covid/.

[21] Prison Reform Trust (2021). CAPPTIVE: prisoners’ health during COVID-19. https://prisonreformtrust.org.uk/publication/capptive-prisoners-health-during-the-covid-19-pandemic/.

[22] Slotboom A-M, Kruttschnitt C, Bijleveld C (2011). Psychological well-being of incarcerated women in the Netherlands: Importation or deprivation?. Punishm Soc.

[23] Steeg S, Mok PLH (2020). Substance misuse disorder linked to high risk of self-harm. Lancet Psychiatry.

[24] Corston BJ (2007). The corston report: a review of women with particular vulnerabilities in the criminal justice system. https://webarchive.nationalarchives.gov.uk/ukgwa/+/http://www.homeoffice.gov.uk/documents/corston-report/.

