# Supplementary material for: Mixed-methods investigation into the prevalence, patterns and determinants of prisoner self-harm during the COVID-19 pandemic in England and Wales compared with prepandemic self-harm (COPE)
Source: BMJ Public Health. 2025 Nov 21;3(2):e002392. doi: 10.1136/bmjph-2024-002392 (PMC12645642; doi:10.1136/bmjph-2024-002392)
Supplement: online supplemental file 1 [file bmjph-3-2-s001.pdf]

Supplementary Material

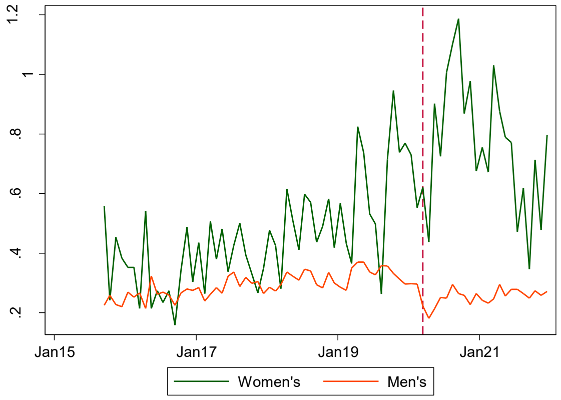

Figure S1: monthly rate of self-harm requiring hospitalisation, per 100 prisoners, men's and women's estates

Table S1: Characteristics of survey participants

**Characteristic**	**N (%)**
Age Range (Years)	
20-29	5 (6)
30-39	22 (25)
40-49	28 (31)
50-59	32 (36)
60-69	1 (1)
Prefer not to say	1 (1)
Sex at Birth	
Female	37 (42)
Male	51 (57)
Prefer not to say	1 (1)
Ethnicity	
White	86 (97)
Ethnic minority people (excluding white minorities)	2 (2)
Prefer not to say	1 (1)
Time in Prison Service (Years)	
1-4	7 (8)
5-9	9 (10)
10-14	7 (8)
15-19	28 (31)
20-24	14 (16)
25-29	10 (11)
30-34	10 (11)
35-40	4 (5)
Prison Category	
Women's	8 (9)
Men's Cat A	6 (7)

Men's Cat B	27 (30)
Men's Cat C	31 (35)
Men's Cat D	11 (12)
Men's Mixed Category	6 (7)

Table S2 summary of policy-related responses from the qualitative consultation, interviews and open questions.

Policy	Described as beneficial for people who self-harm	Discussed by prisoners	Discussed by staff	Details
In-cell phones	Yes	Yes	Yes	Provided access to friends, family, helplines, mental health checks:<br><br>*"I think the in-cell telephony was the biggest thing. I oversaw that project when Covid first started, and we were one of the sites that was identified to kind of rush that through. It was quite stressful but absolutely, yes, I think it was life changing for the men. That contact that they could have with their partner whenever they wanted. Because previously, before that you'd got three phones, you'd got three phones on a landing between sixty prisoners. Could you imagine the kind of fighting you'd have over it?"*<br><br>*"What we did is we also got a lot of telephones in different areas throughout the prison, so that we can ring prisoners in cell. And that was a massive game changer, which is probably one answer to one of the questions earlier, that has really made our lives a lot easier. So now we can do, healthcare can do telephone appointments, education can do check in, we can do mental health wellbeing check in."*
Extra Phone Credit	Yes	No	Yes	Helped prisoners maintain family contact and reduced the likelihood of debt, which can lead to bullying and self-harm<br><br>*"But by having that extra phone credit and that extra money, it stopped them thinking about, well I need to borrow or I need to lend vapes or I need to do this, because I've got the money there. So those prisoners that normally wouldn't have any money and wouldn't go to work because they were under threat of being*

				bullied or getting themselves into debt, we gave them the money to buy what they needed. They never had to borrow or get into debt. And so, in turn, by not getting into debt, not being bullied, self-harm was down"
Removal of Basis Incentives and Earned Privileges	Yes	No	Yes	Removed something seen as punitive<br><br>Enabled ongoing access to TVs. This was seen as beneficial because: it enabled people to distract themselves from self-harming; it enabled staff to communicate with residents via WayOut TV; and it led to less conflict because residents were not angry about their TVs being taken away.<br><br>But, officers/staff felt loss of a behavioural incentive can create discipline problems in prison.<br><br>*"We've learnt through Covid that actually taking a telly as a punishment is not helpful to somebody's mental health, whether they self-harm, whether they take drugs, it's probably going to make them take drugs more because they're bored. So, we moved away from that and we're looking more at a proper incentivised IEP scheme, rather than it being all sanction led."*
Suspension of In-person visits	Mixed	Yes	Yes	From both the PPN consultation and interviews with staff, losing in-person visits was described as the hardest things for prisoners with reduced contact with families seen as likely to lead to more self-harm and worse mental health<br><br>*"Speaking to people it's clear their self-harm is driven by lack of support and the fact that people who are suffering don't have access to their usual outlets. Family, friends, gym, etc."*<br><br>However, it was recognised that in-person visits can be emotionally difficult for some people:<br><br>*"They didn't have to worry so much about what was going on outside anymore. But they still received letters and video visits. But normally when visitors arrive yes there's positive, there's a lot of positive aspects of certainly your mom or dad coming up. But if it's a partner or ex-partner and their partners are saying how*

				difficult it is at home for them with the children, etc. Those messages are much more effective if it's a face to face one. If they go off to a wing, they kind of escape from what's going on outside, they are kind of protected. When the partner comes up and tells them how awful it's been for the kids during the day and little Billy has been suspended from school again, and all the rest of it. Of course, that gives them the stress again and they are feeling guilty about what's happening because they're not able to do anything because they're in the prison."
Introduction of Video 'Purple' visits	Mixed	Yes	Yes	Staff said that 'Purple' visits were beneficial for prisoners and also cheaper, more convenient and less stressful for families<br><br>There were problems with Purple Visits especially technical drops in connectivity, or where visits cut out because of security technology; families struggling to understand the technology; and a limited number of computers/tablets and staff to facilitate their use<br><br>Some staff reported that residents enjoyed being able to 'see into their family home'; others reported prisoners were distressed and downhearted because of this<br><br>Prisoners said Purple Visits could be positive, but were no replacement for in-person visits and technology problems could make them 'worse than nothing'.<br><br>*"Purple visits were good. I was able to see my family home and know I wasn't in any risk of passing on the virus or catching it, but it just isn't the same and never will be [as a physical visit]."*<br><br>*"Video calls freeze with sudden movement. My sister's dog appeared in the back and the call went off. Waited months for this and it was worse than nothing."*
Provision of a Wellbeing Fund	Inconclusive	No	Yes	Staff discussion of the wellbeing fund focused on what was purchased with the money, e.g. electronics, distraction materials, exercise equipment, rather than effects of the funds on self-harm
